# Comparative transcriptomic analysis of endometrial tissue associated with uterine fibroids and endometrial polyps

**DOI:** 10.1038/s41598-026-57099-9

**Published:** 2026-06-08

**Authors:** Lidia Korczyńska, Michalina Dąbrowska, Maria Kulecka, Ewa E. Hennig, Mohamed Ali, Aneta Bałabas, Paweł Czarnowski, Piotr Olcha, Tomasz Łoziński, Antonio Simone Laganà, Jerzy Ostrowski, Michał Ciebiera, Natalia Zeber-Lubecka

**Affiliations:** 1 2nd Department of Obstetrics and Gynecology, Centre of Postgraduate Medical Education, Warsaw, Poland; 2 Department of Experimental Oncology, Laboratory of Cancer Metabolism, Maria Sklodowska-Curie National Research Institute of Oncology, Warsaw, Poland; 3 Department of Gastroenterology, Hepatology and Clinical Oncology, Centre of Postgraduate Medical Education, Warsaw, Poland; 4 Clinical Pharmacy Department, Faculty of Pharmacy, Ain Shams University, Cairo, Egypt; 5 Department of Obstetrics and Gynecology, University of Chicago, Chicago, IL USA; 6 Department of Gynecology and Gynecological Endocrinology, Medical University of Lublin, Lublin, Poland; 7 Institute of Medical Sciences, Medical College of Rzeszow University, Rzeszów, Poland; 8 Unit of Obstetrics and Gynecology, Department of Health Promotion, Mother and Child Care, Internal Medicine and Medical Specialties (PROMISE), “Paolo Giaccone” Hospital, University of Palermo, Palermo, Italy

**Keywords:** Uterine fibroids, Endometrial polyps, Endometrial transcriptome, RNA sequencing, Biomarker discovery, Reproductive medicine, Biological techniques, Biomarkers, Computational biology and bioinformatics, Diseases, Genetics, Medical research, Molecular biology

## Abstract

Uterine fibroids (UFs) and endometrial polyps (EPs) are common benign gynecological conditions with distinct anatomical origins. However, their molecular impact on endometrial function remains poorly understood. This study aimed to descriptively explore shared and condition‑associated transcriptomic patterns within endometrial tissue by analyzing pathological endometrial tissue (PET) and adjacent macroscopically normal endometrial tissue (NET), sampled from areas without visible lesions in uteri affected by UFs or EPs. Paired PET and NET samples were collected during hysteroscopy from women diagnosed with UFs or EPs, with RNA‑sequencing performed on samples derived from premenopausal and perimenopausal patients. RNA sequencing and differential gene expression analysis were performed using DESeq2, followed by functional enrichment via GO and Reactome databases. Only nine differentially expressed genes (DEGs) were identified when comparing NET samples associated with UFs and EPs. In contrast, comparison of PET samples between UFs and EPs revealed 398 DEGs. Furthermore, PET versus NET comparisons demonstrated a pronounced quantitative difference between conditions, with 3163 DEGs identified in EPs and only 77 DEGs in UFs. EP-associated DEGs were enriched in immune activation, epithelial remodeling, and hormonal signaling pathways, while UF-associated DEGs reflected localized changes in inflammation, oxidative stress, and extracellular matrix remodeling. A small subset of genes (*FOSB*,* DPP4*,* TM4SF4*,* DNER*,* AOX1*, and *PAEP*) was consistently dysregulated across both conditions, suggesting shared transcriptional patterns associated with altered tissue contexts. This exploratory study provides insights into transcriptomic features of endometrial tissue associated with UFs and EPs. The findings highlight both shared and context‑dependent transcriptional patterns and identify candidate genes that warrant further investigation in future, independent studies aimed at elucidating endometrial responses to benign uterine pathology.

**Supplementary Information:**

The online version contains supplementary material available at 10.1038/s41598-026-57099-9.

## Introduction

Uterine fibroids (UFs) and endometrial polyps (EPs) are distinct yet frequently coexisting benign uterine pathologies, each characterized by a unique molecular background. EPs are benign proliferations of the endometrial lining, composed of glands, stroma, and blood vessels, forming pedunculated or sessile structures protruding into the uterine cavity^1^. Their etiology is complex, involving both hormonal and molecular factors. A key role in their pathogenesis is played by the overexpression of endometrial aromatase, leading to locally increased estrogen concentrations^2^.

Polyps exhibit features of clonal proliferation, suggesting the involvement of somatic genetic mutations^1^. Mutations have been identified in genes commonly associated with endometrial cancer, such as *KRAS*,* PTEN*,* TP53*,* PIK3CA*,* AKT1*,* ARID1A*, and *FBXW7*, although they occur at low allelic frequencies^3^. These mutations are primarily localized to the epithelial component of polyps, which may explain their potential for neoplastic transformation, particularly in postmenopausal women or those treated with tamoxifen^4^. Additionally, transcriptomic analysis has revealed disruptions in Wnt signaling pathways and smooth muscle regulation, which may contribute to uncontrolled growth and clinical symptoms such as abnormal bleeding and infertility^5^.

On the other hand, UFs are benign tumors originating from the smooth muscle layer of the uterus (myometrium), rather than from the endometrial lining^6^. Although fibroids arise from the myometrium, their presence influence the molecular environment and function of the overlying endometrium^7,8^. They are among the most common benign tumors of the female reproductive tract. Their prevalence increases with age and varies across populations, with estimates suggesting that up to 70% of women approaching menopause may develop fibroids^6^. Women of reproductive age present a particular therapeutic challenge, as clinically apparent UFs occur in 25–50% of this group, and approximately 25% require treatment^9^. Gonadal steroids, primarily estrogens and progestogens, play a central role in UF pathogenesis^10^. Genome-wide association studies (GWAS) have identified polymorphisms in genes such as *ODFC3*,* BET1L*, *RIC8A*,* SIRT3*,* SLK*,* OBFC1*,* TNRC6B*,* FASN*, and *HMGA2*^11^. The most frequently observed point mutation is in the *MED12* gene (Xq13), found in up to 70% of cases^12^. This gene encodes a subunit of RNA polymerase II mediator complex, and its mutations may influence not only fibroid formation but also clinical features such as tumor size, location, and multiplicity^12^. Other genes with reported mutations include *CAPRIN1*,* DCN*, and *AHR*, which are involved in cell cycle regulation and tumor suppression^11^.

Despite clear differences in the etiopathogenesis and molecular profiles of UFs and EPs, the mechanisms underlying their coexistence and their impact on endometrial function remain poorly understood. Previous studies have largely focused on pathological lesions themselves, often without comparison to adjacent endometrial tissue within pathology‑affected uteri. Importantly, although UFs originate from the myometrium and EPs from the endometrial lining, both conditions are known to modulate the molecular environment of the endometrium. To address this gap, the present study includes both pathological endometrial tissue (PET) and adjacent macroscopically normal endometrial tissue (NET), defined as endometrium without visible lesions at hysteroscopy and interpreted throughout the study as macroscopically non‑lesional but pathology‑associated tissue rather than biologically healthy endometrium. This approach enables a more comprehensive assessment of local gene expression patterns associated with distinct uterine pathological contexts.

## Materials and methods

### Ethics statement

The study was approved by the Bioethics Committee at the Centre of Postgraduate Medical Education in Warsaw, Poland (project ID: 63/2022). It was conducted in accordance with the ethical guidelines of the 1975 Declaration of Helsinki, as revised in 1964. Written informed consent for participation was obtained from all participants prior to inclusion in the study. The study was carried out in collaboration with the 2nd Department of Obstetrics and Gynecology at the Warsaw Institute of Women’s Health and the Department of Gastroenterology, Hepatology, and Clinical Oncology at the Centre of Postgraduate Medical Education in Warsaw.

### Subjects and samples collection

All enrolled participants were Caucasian women aged ≥ 18 years who underwent hysteroscopy for standard gynecological indications. Between June 2022 and July 2023, a total of 29 patients were recruited, including women diagnosed with UFs and those with EPs. Exclusion criteria included pregnancy or lactation, chronic immunosuppression, ongoing or recent oncological treatment, use of hormonal contraception or menopausal hormone therapy, hormonal treatment for endometrial hyperplasia within one month prior to hysteroscopy, use of a hormonal intrauterine device, abnormal cervical cytology, and antibiotic therapy within 30 days preceding the procedure. Recruitment was not restricted by menopausal status and included premenopausal, perimenopausal, and postmenopausal women. For transcriptomic analyses, however, only samples obtained from premenopausal and perimenopausal patients (*n* = 27) were finally included to minimize confounding effects related to postmenopausal hormonal deprivation and age‑associated changes in endometrial gene expression.

**Fig. 1:**
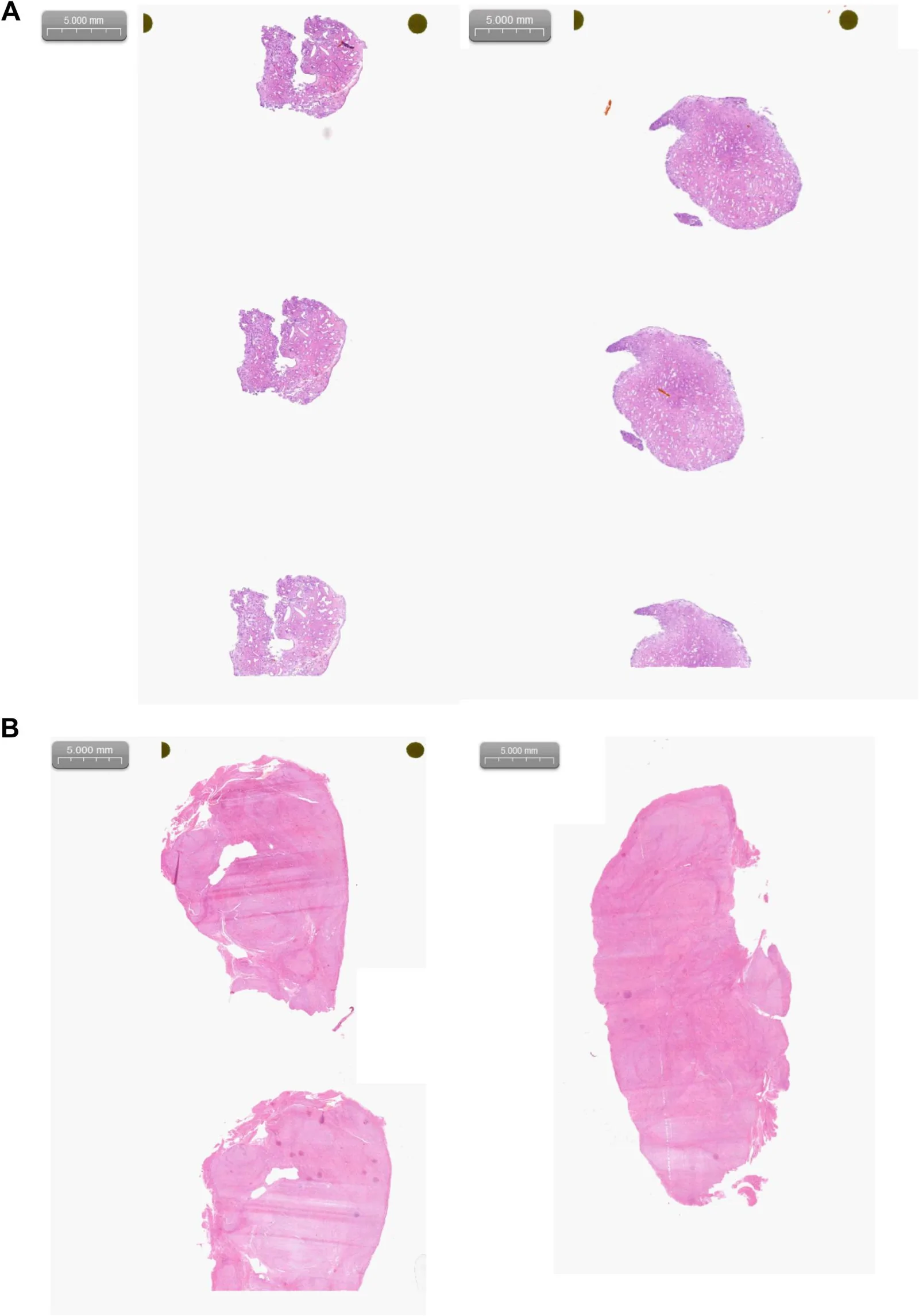
Histopathological images of (**A**) endometrial polyps and (**B**) uterine fibroids. Panel (**A**) shows the characteristic architecture of an endometrial polyp, with endometrial glands embedded in a fibrotic stroma of variable density. Panel (**B**) presents a uterine fibroid, composed of interlacing bundles of smooth muscle cells without cytological atypia. Both panels stained with hematoxylin and eosin (H&E); original magnification ×200.

During hysteroscopy, endometrial biopsies were collected from sites displaying pathological changes or, in the case of UFs, from endometrium located directly above myometrial lesions deforming the uterine cavity. NET was sampled at a distance of at least 1 cm from visible pathology. In the context of UFs, PET refers to endometrial tissue sampled in close proximity to underlying fibroids, whereas fibroid tissue itself was not collected or analyzed. Both UF and EP groups were recruited under identical clinical indications and scheduling conditions for hysteroscopy, without differential selection based on menstrual timing. Specimens intended for RNA‑sequencing were immediately placed in RNAlater Stabilization Solution (Invitrogen, Thermo Fisher Scientific, USA), snap‑frozen, and stored at − 80 °C. Samples designated for histopathological evaluation were fixed in 10% formaldehyde, sectioned, and stained with hematoxylin and eosin. Histological assessment was performed by an experienced pathologist, and representative findings are shown in Fig. 1.

### RNA isolation, sequencing, and data analysis

Total RNA was extracted from tissue samples using the mirVana™ PARIS™ RNA and Native Protein Purification Kit (Thermo Fisher Scientific, Waltham, MA, USA), following the manufacturer’s instructions. RNA concentration and purity were assessed using a NanoDrop™ 2000 Spectrophotometer (Thermo Fisher Scientific), and RNA integrity was evaluated with an Agilent RNA 6000 Nano Kit on a 2100 Bioanalyzer (Agilent Technologies, Santa Clara, CA, USA). All samples retained for RNA‑sequencing exhibited high RNA quality, with RNA integrity numbers (RIN) ≥ 8, and were stored at − 80 °C until library preparation.

RNA-Seq libraries were prepared and sequenced as outlined in^13^. Signal processing and base calling were performed using Torrent Suite software version 5.10 [https://github.com/iontorrent/TS]. Sequencing reads were aligned to the hg19 reference genome, as this genome build is supported by the Ion AmpliSeq™ Transcriptome Human Gene Expression Kit (v1) and the ampliSeqRNA analysis pipeline used in this study. Gene‑level read counts were generated using HTSeq‑count version 0.615, ensuring consistency across all samples^14^, using the default settings. Library size differences were accounted for through DESeq2 normalization, and gene‑wise dispersion was estimated using moderated estimation as described by Love et al.^15^. Differential gene expression analysis was conducted with DESeq2 version 1.38^15^. Analyses were performed at the sample level, treating each sequenced tissue sample as one observational unit. Patient identity was not included as a blocking factor in the design formula due to limited cohort size and incomplete PET-NET pairing. Default DESeq2 independent filtering was applied, and log_2_FoldChange (log_2_FC) shrinkage was not performed. Genes with an adjusted p-value (padj) of < 0.05 were classified as differentially expressed. During data analysis, standard DESeq2 diagnostic outputs, including MA plots, were examined to assess data quality and variability. Principal component analysis (PCA) was used for exploratory visualization of global transcriptomic variability, and group differences were assessed using permutational multivariate analysis of variance (PERMANOVA). Functional enrichment analysis of differentially expressed genes (DEGs) was performed using the ClueGO plugin (version 2.5.1) within Cytoscape (version 3.6.1)^14^, covering Gene Ontology Biological Process (BP), Molecular Function (MF), Cellular Component (CC), Immune Process categories, and Reactome pathways^16^. P‑values were adjusted for multiple testing using the Benjamini-Hochberg correction, with a significance threshold set at < 0.05.

## Results

### Patients’ characteristics

The transcriptomic analyses included a total of 27 patients, comprising 15 women diagnosed with UFs and 12 with EPs. The median age was 44.5 years (range: 34–53 years) in the UF group and 47.0 years (range: 31–55 years) in the EP group (Table 1). In both groups, five patients reported no prior pregnancies. At least one childbirth was reported by seven women in the UF group and eight in the EP group. A history of miscarriage was recorded in five UF patients and three EP patients. Previous infertility treatment was reported exclusively in the UF group (*n* = 4). Among women with UFs, one patient was diagnosed with polycystic ovary syndrome and one with endometriosis; these comorbidities were not observed in the EP group. Vitamin D supplementation was more frequently reported in the UF group (*n* = 10) than in the EP group (*n* = 3). Antibiotic use within 1–6 months preceding surgery was noted in six women with UFs and three with EPs. Previous medical procedures or surgical interventions, including gynecological procedures and abdominal operations, were reported by 12 patients in the UF group and five in the EP group.

**Table 1:** Demographic and clinical characteristics of patients included in transcriptomic analyses.

	Uterine fibroids *N* = 15 (55.6%)	Endometrial polyps *N* = 12 (44.4%)
Median age; years [range]	44.5 [34–53]	47.0 [31–55]
No pregnancy	5	5
Given birth	7	8
Miscarriage	5	3
Infertility treatment	4	0
History of inflammatory conditions of the reproductive organs	5	3
Polycystic ovary syndrome	1	0
Endometriosis	1	0
Vitamin D supplementation	10	3
Antibiotic use within 1–6 months before surgery	6	3
Medical procedures/operations	12	5

### Transcriptomic profiling

To perform transcriptomic profiling, total RNA was isolated from paired PET and NET samples obtained from premenopausal and perimenopausal women undergoing hysteroscopy. Following RNA quality assessment (including RIN evaluation) and downstream bioinformatic quality control, a subset of collected samples fulfilling predefined quality criteria was retained for analysis. The final RNA‑sequencing dataset comprised 47 endometrial samples, including 26 samples from the UFs group (15 NET and 11 PET) and 21 samples from the EPs group (12 NET and 9 PET).

**Fig. 2:**
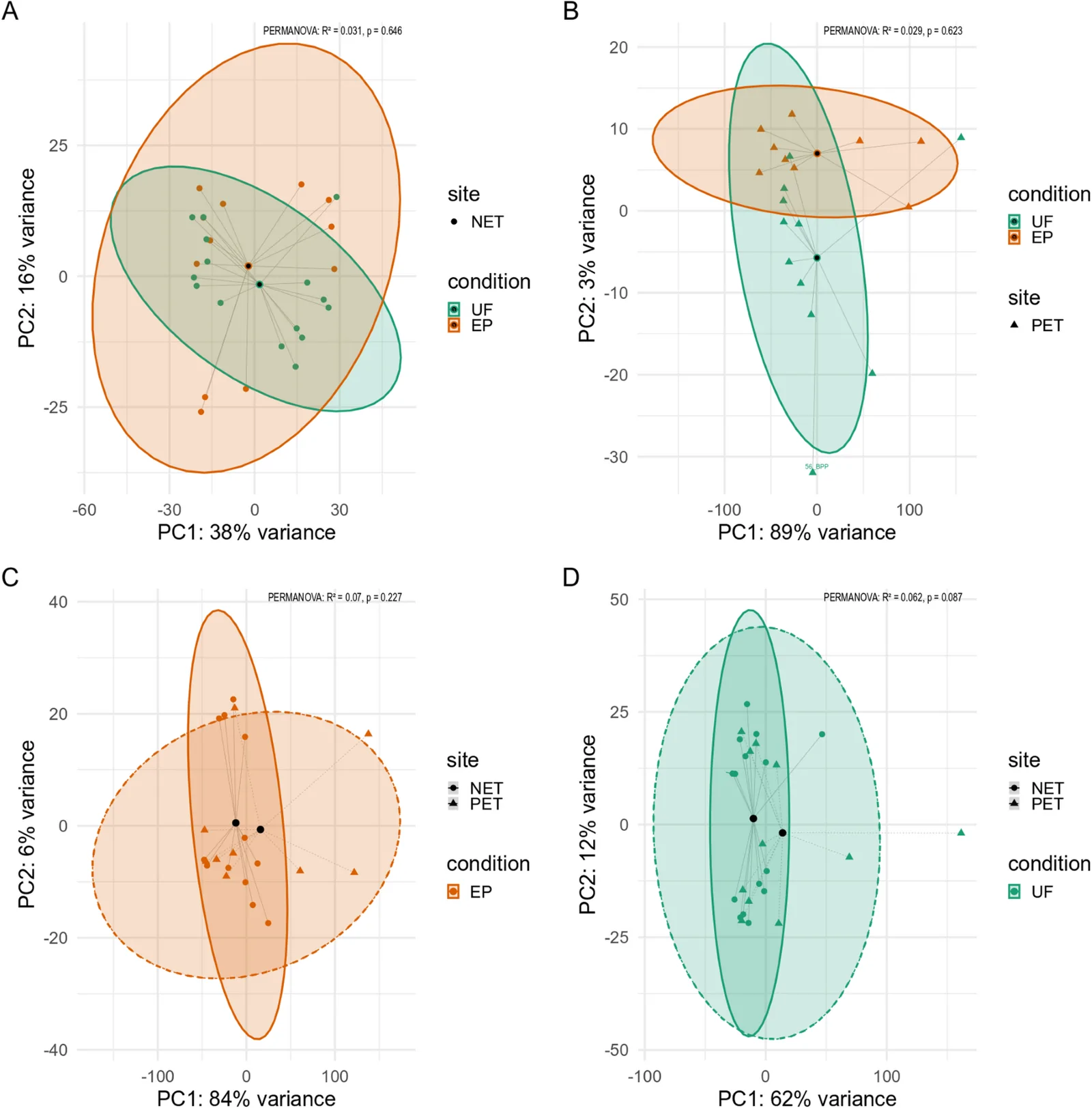
Exploratory principal component analysis (PCA) of transcriptomic profiles. PCA was performed to visualize global transcriptomic variability across samples. (**A**) Macroscopically non lesional endometrial tissue (NET): uterine fibroids (UF, *n* = 15) vs. endometriosis (EP, *n* = 12). (**B**) Pathologically altered endometrial tissue (PET): UF (*n* = 11) vs. EP (*n* = 9). (**C**) EP group: PET (*n* = 9) vs. NET (*n* = 12). (**D**) UF group: PET (*n* = 11) vs. NET (*n* = 15). Colors indicate pathology (UF vs. EP), and point shapes indicate tissue type (NET vs. PET).

PCA showed no significant differences in community composition between UF and EP conditions at either site, with PERMANOVA explaining a limited proportion of variance (NET: R² = 0.031, p-value = 0.646; PET: R² = 0.029, p-value = 0.623) (Fig. 2A and B). Similarly, no statistically significant separation was observed between NET and PET sites within EP or UF conditions (EP: R² = 0.070, p-value = 0.227; UF: R² = 0.062, p-value = 0.087) (Fig. 2C and D), despite PC1 accounting for a large fraction of total variance (62–89%).

**Fig. 3:**
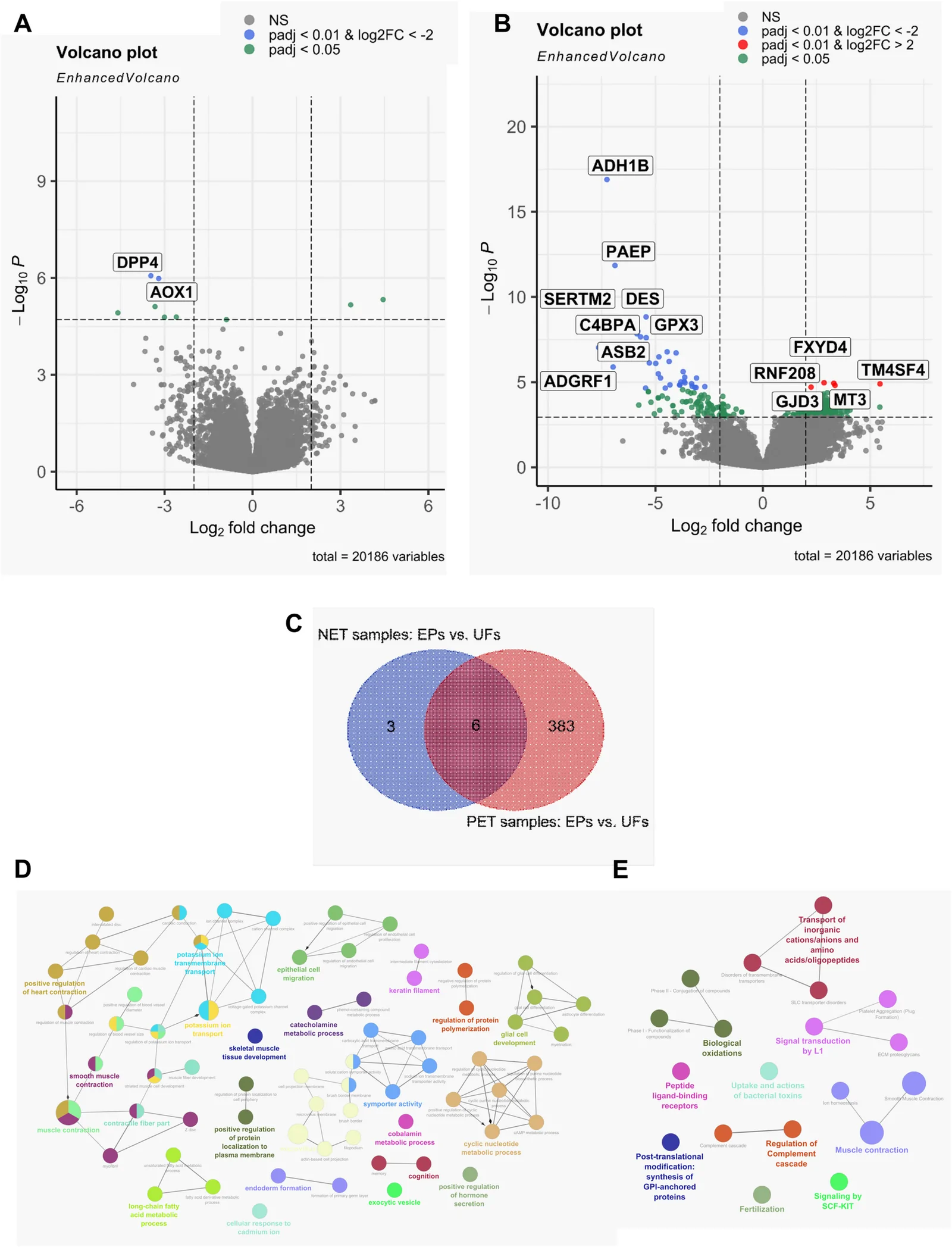
Volcano plots showing gene expression differences: (**A**) In macroscopically non‑lesional endometrial tissues (NET), comparing UFs vs. EPs. (**B**) In pathologically altered endometrial tissues (PET), comparing UFs vs. EPs. (**C**) Venn diagram illustrating the number of differentially expressed genes (DEGs) unique to NET comparisons (left), unique to PET comparisons (right), and shared between NET and PET analyses (intersection). (**D**) and (**E**) The top overrepresented pathways identified through ClueGO functional enrichment analysis of PET unique DEGs set [**D**: Biological Process (BP), Molecular Function (MF), and Cellular Component (CC) categories of Gene Ontology (GO); (**E**): Reactome pathways)]. The name of the group is by default the most significant term of the group.

Pairwise comparisons between EP and UF groups revealed nine and 398 DEGs (padj < 0.05) depending on the tissue type (NET and PET, respectively). Among NET samples, seven out of nine genes (77.8%), including *PAEP* (log_2_FC -4.59; padj = 0.03), *AOX1* (log_2_FC -3.20; padj = 0.008), *DPP4* (log_2_FC -3.47; padj = 0.008), *FOSB* (log_2_FC -3.32; padj = 0.02), *DNER* (log_2_FC -3.00; padj = 0.03), LOC100505989 (log_2_FC -2.60; padj = 0.03) and *ERN1* (log_2_FC -0.89; padj = 0.034) were higher expressed in UF than EP tissues. In turn, the expression of two out of nine genes (22.2%), *SOSTDC1* (log_2_FC 3.34; padj = 0.02) and *TM4SF4* (log_2_FC 4.45; padj = 0.02), was higher in EP tissues (Fig. 3A Volcano plot, Supplementary Table 1). In contrast, within PET tissues, 106 out of 389 genes (27.2%) were more highly expressed in UF samples and 283 (72.8%) in EP samples (Fig. 3B Volcano plot, Supplementary Table 1). Among the UF-upregulated genes with the lowest padj were *ADH1B* (log_2_FC -8.55; padj = 2.16 × 10⁻¹³), *PAEP* (log_2_FC -7.08; padj = 1.20 × 10⁻⁸), *GPX3* (log_2_FC -6.05; padj = 8.34 × 10⁻⁶), *SERTM2* (log_2_FC -5.84; padj = 2.20 × 10⁻⁵), and *DES* (log_2_FC -5.73; padj = 3.41 × 10⁻⁵). Similarly, among the EP-upregulated genes, the most statistically significant included *RNF208* (log_2_FC 2.85; padj = 0.0074), *FXYD4* (log_2_FC 3.31; padj = 0.0076), *TM4SF4* (log_2_FC 5.46; padj = 0.0076), *MT3* (lo_g_2FC 3.36; padj = 0.0084), and *GJD3* (log_2_FC 2.25; padj = 0.0097) (Supplementary Table 1).

A Venn diagram was used to illustrate the overlap of DEGs between EPs vs. UFs comparisons conducted separately within NET and PET samples. A total of six genes, *FOSB*,* DPP4*,* TM4SF4*,* DNER*,* AOX1*, and *PAEP*, were commonly differentially expressed in both tissue types. In contrast, three genes, *SOSTDC1*,* ERN1*, and *LOC100505989*, were uniquely differentially expressed in the NET comparison. Remarkably, 383 DEGs were unique for PET samples (Fig. 3C, Supplementary Table 1).

Next, we performed functional enrichment analysis of 383 DEGs that significantly differentiate UF and EP tissues uniquely to PET samples. We identified 62 signaling pathways or biological processes based on GO database categories (including: *muscle contraction*,* microvillus*,* potassium ion transport*,* microvillus membrane*,* epithelial cell migration*,* keratin filament*,* filopodium*,* and long-chain fatty acid metabolic process*, (padj < 0.05; Fig. 3D; (Supplementary Table 2), as well as 19 pathways from the Reactome Pathway database (including: *Biological oxidations*,* Fertilization*,* Signaling by SCF-KIT*,* Post-translational modification - synthesis of GPI-anchored proteins*,* Uptake and actions of bacterial toxins*,* and ECM proteoglycans* (padj < 0.05; Fig. 3E; (Supplementary Table 2). Due to the low number of NET-specific genes and the overlap being limited to genes shared with PET samples, the functional enrichment analysis did not reveal any significant pathways.

**Fig. 4:**
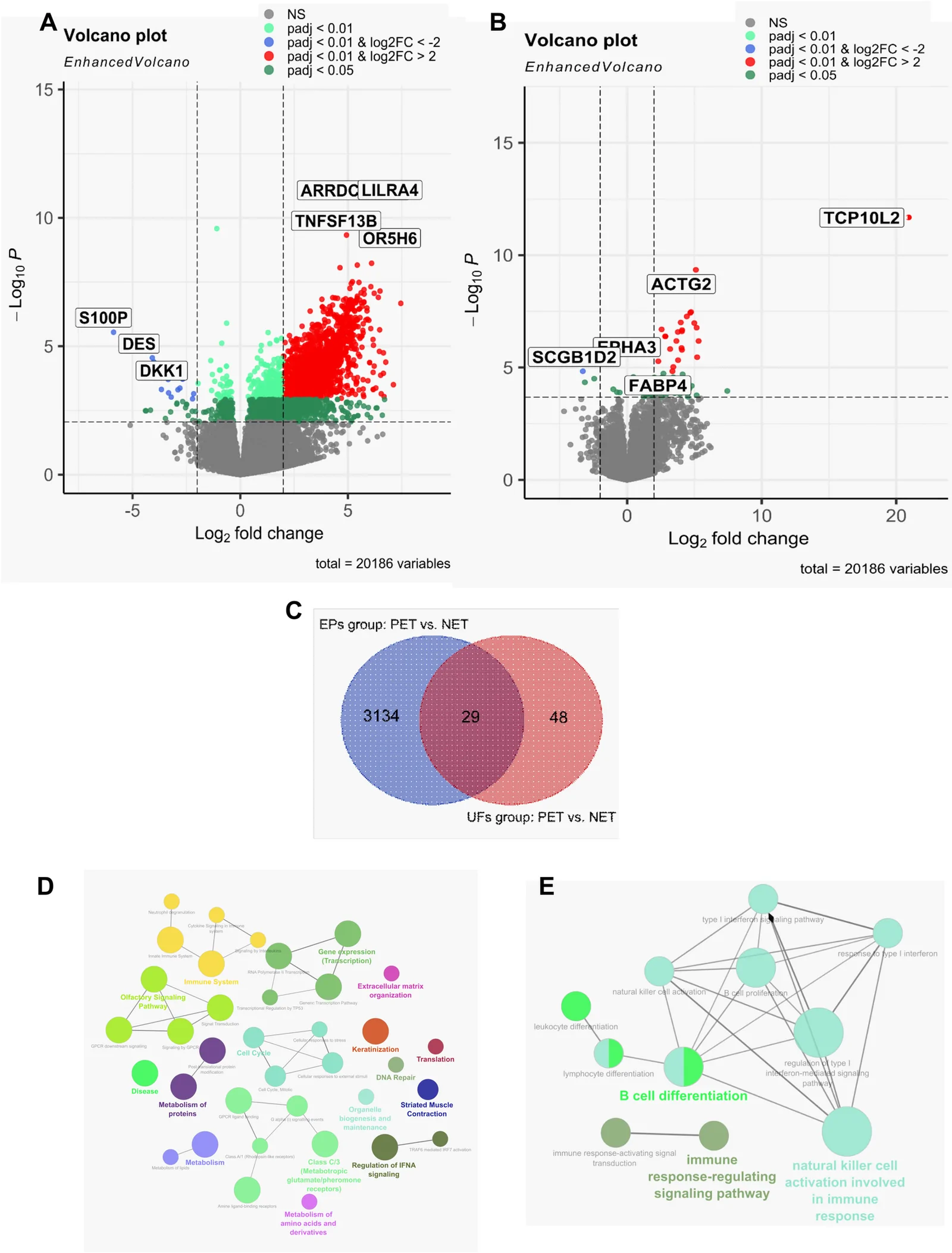
Volcano plots showing gene expression differences between pathologically altered (PET) and macroscopically non‑lesional (NET) endometrial tissues: (**A**) Endometrial polyps (EPs). (**B**) Uterine fibroids (UFs). (**C**) Venn diagram showing the number of differentially expressed genes (DEGs) unique to PET vs. NET comparisons in EPs (left), unique to PET vs. NET comparisons in UFs (right), and genes shared between both pathological contexts (intersection). (**D**) and (**E**) The top overrepresented pathways identified through ClueGO functional enrichment analysis of EPs unique DEGs set [**D**: Reactome pathways. (**E**): Gene Ontology (GO) Immune Processes]. The name of the group is by default the most significant term of the group.

Finally, we identified DEGs (padj < 0.05) that significantly differentiated PET and NET tissues, with analyses performed separately for EPs and UFs. In the EP group, we identified 3163 DEGs, of which 2932 (92.7%) and 231 (7.3%) were upregulated in PET and NET samples, respectively (Supplementary Table 3). Among the most significantly upregulated genes in EP-PET samples were *ARRDC3-AS1* (log_2_FC 4.94; padj = 4.14 × 10⁻⁶), *OR5H6* (log_2_FC 6.10; padj = 3.06 × 10⁻⁵), *LILRA4* (log_2_FC 5.43; padj = 3.06 × 10⁻⁵), *TNFSF13B* (log_2_FC 4.63; padj = 3.09 × 10⁻⁵), *LOC253573* (log_2_FC 6.07; padj = 7.87 × 10⁻⁵), *LOC100506497* (log_2_FC 5.82; padj = 7.87 × 10⁻⁵), *ATP1B4* (log_2_FC 5.75; padj = 7.87 × 10⁻⁵), and *IL24* (log_2_FC 5.51; padj = 7.87 × 10⁻⁵) (Fig. 4A, Supplementary Table 3). In turn, the most statistically significant DEGs among the NET samples from the EP group included *MRPS21* (log_2_FC -1.09; padj = 4.14 × 10⁻⁶), *CHD9* (log_2_FC -0.63; padj = 1.83 × 10⁻⁴), *S100P* (log_2_FC -5.88; padj = 2.87 × 10⁻⁴), *NUP88* (log_2_FC -0.86; padj = 4.37 × 10⁻⁴), *DES* (log_2_FC -4.08; padj = 9.36 × 10⁻⁴), *CBWD1* (log_2_FC -1.37; padj = 1.09 × 10⁻³), *DKK1* (log_2_FC -4.01; padj = 1.16 × 10⁻³), and *EIF2A* (log_2_FC -0.64; padj = 1.40 × 10⁻³) (Supplementary Table 3).

In the UF group, differential expression analysis revealed 77 DEGs, with the vast majority (69 genes; 89.6%) upregulated in PET samples and only a small subset (8 genes; 10.4%) upregulated in NET samples (Fig. 4B, Supplementary Table 3). Among the PET-upregulated genes with the highest statistical significance were *LOC152024* and *TCP10L2* (log_2_FC 20.92; padj = 1.82 × 10⁻⁸), *ACTG2* (log_2_FC 5.11; padj = 2.60 × 10⁻⁶), *LY6H* (log_2_FC 4.75; padj = 1.29 × 10⁻⁴), *DES* (log_2_FC 4.70; padj = 1.29 × 10⁻⁴), *IL17B* (log_2_FC 4.43; padj = 1.55 × 10⁻⁴), *PRLHR* (log_2_FC 4.99; padj = 2.28 × 10⁻⁴), and *ITGB1BP2* (log_2_FC 3.99; padj = 2.28 × 10⁻⁴). Conversely, NET samples showed upregulation of a distinct set of genes, including *POLR3B* (log_2_FC -0.80; padj = 1.34 × 10⁻³), *DIP2B* (log_2_FC -0.98; padj = 4.02 × 10⁻³), *SCGB1D2* (log_2_FC -3.29; padj = 9.45 × 10⁻³), *PTGS2* (log_2_FC -2.44; padj = 1.61 × 10⁻²), *MUC5B* (log_2_FC -3.14; padj = 2.03 × 10⁻²), *NONO* (log_2_FC -1.03; padj = 2.99 × 10⁻²), *SPRYD7* (log_2_FC -0.75; padj = 3.57 × 10⁻²), and *PDE12* (log_2_FC -0.55; padj = 3.62 × 10⁻²), *ITGA5* (log_2_FC 1.41; padj = 0.04); *HSPB1* (log_2_FC 1.32; padj = 0.02), *SLC7A3* (log_2_FC 2.87; padj = 0.04), *LRRC2* (log_2_FC 2.59; padj = 0.01) (Supplementary Table 3). The marked difference in DEG counts between EP and UF comparisons highlights substantial quantitative heterogeneity in transcriptomic responses observed across distinct pathological contexts.

Next, we identified genes that were either shared or unique to PET vs. NET comparisons performed separately for EP and UF groups. A total of 29 genes, including *ACTG2*,* CNN1*,* HLA-G*,* CASP14*,* FIBIN*,* NONO*,* LINC00628 (TOB1-AS1)* were commonly differentially expressed in both comparisons. In contrast, 3134 genes (*TRIM33*,* SOD2*,* FOXP3*,* MMP9*,* DKK1*) were uniquely identified in the EP group, while 48 genes (*IL17B*,* PTGS2*,* MUC5B*,* ITGA5*,* HSPB1*,* SLC7A3*,* LRRC2*) were exclusive to the UF group.

Functional enrichment analysis of DEGs that significantly differentiate PET and NET tissues in gene sets unique to EP and UF samples. For the 3134 EP-specific genes, we identified 194 pathways based on GO database BP, MF, and CC categories (Supplementary Fig. 1; Supplementary Table 4). The most significantly enriched terms (padj < 0.05) included the following GO pathways: *olfactory receptor activity; G-protein coupled receptor activity; intermediate filament; keratinization; extracellular exosome; extracellular vesicle; metal ion binding as well as mitochondrion*. We identified also 13 pathways for this set of genes based on the GO Immune Process, which included, among others, *natural killer cell activation involved in immune response; regulation of type I interferon-mediated signaling pathway; B cell differentiation; lymphocyte differentiation; and response to type I interferon* (Fig. 4E; Supplementary Table 4). Using the Reactome Pathway database, we identified 36 significantly enriched pathways (padj < 0.05). The most enriched terms included: *Olfactory Signaling Pathway; GPCR downstream signaling; Signaling by GPCR; Signal Transduction; Keratinization; Metabolism of proteins; Immune System; Post-translational protein modificatio*n as well as *Class C/3 (Metabotropic glutamate/pheromone receptors)* (Fig. 4D; Supplementary Table 4). The low number of UF-specific genes and minimal overlap with other sets resulted in no significant pathways being identified through functional enrichment analysis.

## Discussion

UFs and EPs are among the most common benign gynecological conditions, yet the molecular mechanisms underlying their development and impact on endometrial function remain incompletely understood^1,17^. Previous transcriptomic studies have largely focused on pathological tissues, often overlooking comparisons with normal endometrium^8^. To address this gap, our preliminary study included macroscopically non‑lesional, pathology‑associated endometrial tissue as an internal reference, enabling a more comprehensive analysis of gene expression differences.

To explore subtle transcriptional differences in macroscopically non‑lesional endometrial tissue associated with distinct pathologies, we compared NET samples from women with UFs and EPs. This analysis revealed only nine DEGs, which reflects a limited number of transcriptomic differences between NET samples associated with UFs and EPs. Among these, three genes, *SOSTDC1*,* ERN1*, and *LOC100505989*, were exclusively dysregulated in NET and not detected in PET comparisons. *SOSTDC1*^18^ and *ERN1*^19^ are involved in signaling and stress-response pathways relevant to endometrial receptivity, while *LOC100505989*, despite limited annotation, appears to be regulated in a tissue-specific manner and may be relevant to UF-associated endometrial changes. These observations resemble transcriptomic patterns previously described for proliferative and mid‑secretory endometrial phases in fertile women^20^, although direct phase attribution was not possible in the present cohort. Specifically, the referenced study reported extensive differential expression of lncRNAs, miRNAs, and mRNAs, with enrichment in pathways related to cell adhesion, metabolism, and FoxO signaling. These pathways are closely associated with endometrial receptivity and may partially overlap with the signaling and stress‑related mechanisms identified in NET samples associated with uterine fibroids and endometrial polyps in the present study.

Previous targeted studies have demonstrated context‑dependent expression of proliferation‑ and apoptosis‑related markers in EPs, particularly in postmenopausal women and in association with metabolic factors^21^. Compared to NET, PET samples demonstrated a markedly broader transcriptional response, with distinct gene expression profiles observed in UF- and EP-associated endometrial tissues. In UF-PET samples, upregulated genes were primarily associated with oxidative stress regulation (*ADH1B*,* GPX3*), cytoskeletal organization and structural remodeling (*DES*)^22–24^. EP-PET samples exhibited increased expression of genes involved in epithelial dynamics, ion transport (*FXYD4*), and angiogenesis (*MT3*), reflecting active tissue reorganization^25,26^. Functional enrichment analysis of PET-specific DEGs revealed overrepresentation of pathways related to muscle contraction, epithelial cell migration, and biological oxidations. In turn, Reactome analysis further highlighted processes such as synthesis of GPI-anchored proteins and ECM proteoglycans, which are essential for cell signaling, adhesion, and endometrial remodeling. A subset of genes, *FOSB*,* DPP4*,* TM4SF4*,* DNER*,* AOX1*, and *PAEP*, was consistently dysregulated in both NET and PET samples, indicating molecular features shared across UF- and EP-associated endometrial tissue. These genes are functionally linked to stress response (*FOSB*), immune regulation and cell adhesion (*DPP4*)^27,28^, epithelial proliferation and differentiation (*TM4SF4*,* DNER*)^29,30^, oxidative metabolism (*AOX1*)^20^, and reproductive processes (*PAEP*)^31,32^. Their concurrent expression in both tissue types highlights conserved transcriptional features that may be involved in endometrial remodeling under pathological conditions. Previous studies have also reported transcriptional alterations in endometrial tissue associated with UFs and EPs. For example, differential expression of prokineticin pathway genes and *HOXA10* has been observed, with *PROKR1* upregulated in both conditions, and *PROK1*,* PROKR2*, and *HOXA10* downregulated in UF patients compared to healthy controls^33^. These changes may contribute to impaired endometrial receptivity and help explain infertility often observed in affected individuals.

The comparison between PET and NET allows for a closer look at how localized uterine pathology affects the surrounding molecular environment. While differences in gene expression between these tissue types may be expected, the scale and specificity of these changes remain insufficiently described, especially when considering distinct pathological conditions such as UFs and EPs. In this study, the number of DEGs was substantially higher in EP samples than in UFs. This disparity may stem from differences in tissue origin and biological activity: EPs arise directly from the endometrial lining and are characterized by active epithelial and immune processes, which can trigger broader transcriptomic shifts. In contrast, UFs develop within the myometrial layer and influence the endometrium more indirectly, resulting in a narrower range of gene expression changes. Variations in cellular composition and local signaling may further contribute to the observed differences.

The UF‑associated endometrial transcriptomic profile revealed a focused set of genes associated with biological processes that may reflect endometrial responses to fibroid‑related uterine pathology. Several of these genes are involved in inflammatory signaling and immune modulation, such as *IL17B*, which activates NF-κB via its receptor IL17RB, promoting cytokine expression and potentially inducing cellular senescence within the uterine microenvironment^34^. PTGS2 (COX-2), a well-known mediator of prostaglandin synthesis, further supports inflammatory and angiogenic activity, both of which are implicated in fibroid growth and maintenance^35^. Another group of UF-associated genes reflects alterations in cellular metabolism and stress response. *SLC7A3*, encoding a transporter of cationic amino acids, has been proposed as a therapeutic target due to its role in supporting proliferative activity in fibroid cells^36^. HSPB1, a small heat shock protein, may enhance cellular survival under oxidative stress, contributing to the resilience of fibroid tissue in adverse conditions^37^. Genes such as *ITGA5*^38^ and *MUC5B*^39^ point to extracellular matrix remodeling and mucosal changes, processes central to fibroid development. ITGA5, an integrin subunit, facilitates cell adhesion and matrix interactions, while *MUC5B*, a mucin gene, may influence local immune responses and tissue architecture. Although *LRRC2* remains less well characterized, its potential role in intracellular signaling suggests it may contribute to the altered regulatory landscape observed in fibroid tissue^40^. Hosseini et al.^41^ conducted an integrative multi-omics analysis to identify molecular links between UFs and recurrent implantation failure. By combining gene expression, DNA methylation, and co-expression network data, they identified three key genes, *EDNRB*,* BIRC3*, and *TRPC6*, as shared molecular candidates across both conditions. These genes are implicated in apoptosis regulation, cellular repair, and signal transduction, and may contribute to abnormal tissue growth and impaired endometrial receptivity. Their findings suggest potential therapeutic targets that could be explored to address both fibroid-related pathology and implantation failure. In turn, Kim et al.^42^ applied a transcriptome-wide association study (TWAS) to prioritize biologically relevant susceptibility genes for UFs by integrating GWAS and eQTL data. Their analysis identified nine significant TWAS genes, including two novel candidates, *RP11-282O18.3* and *KBTBD7*, potentially involved in fibroid pathogenesis. Functional enrichment revealed a strong association with immune system processes, suggesting that immune-related signaling may play a central role in fibroid development. Furthermore, chemical-gene interaction analysis highlighted five toxic compounds linked to TWAS genes, pointing to possible environmental contributors to fibroid etiology.

In contrast to the limited number of DEGs identified in the UF group, the comparison between PET and NET samples in EP patients revealed a substantially broader transcriptomic shift, with 3163 DEGs. This extensive gene set enabled robust functional enrichment analysis, highlighting a diverse array of biological processes and signaling pathways specifically associated with EPs. The EP-specific transcriptomic profile was enriched in pathways related to epithelial differentiation, cytoskeletal organization, vesicle trafficking, mitochondrial function, and metal ion binding, consistent with active tissue remodeling and metabolic adaptation. Immune-related processes were particularly prominent, including natural killer cell activation, type I interferon signaling, B cell and lymphocyte differentiation, and response to interferon, indicating a complex and engaged immune microenvironment. Reactome analysis further revealed enrichment in signaling cascades such as GPCR signaling, keratinization, protein metabolism, and post-translational modifications, underscoring the dynamic and hormonally responsive nature of EP-associated endometrial tissue. Notably, enrichment of terms such as olfactory receptor activity and broad GPCR‑related pathways should be interpreted with caution, as these categories are frequently overrepresented in bulk RNA‑seq datasets due to the large size and expression characteristics of olfactory receptor gene families, which constitute a major subclass within the GPCR superfamily, rather than reflecting tissue‑specific biological functions.

Representative genes within this group, such as *TRIM33*^43^, *FOXP3*^44^, *MMP9*^45^, and *DKK1*^46^, reflect key aspects of hormonal regulation, immune tolerance, extracellular matrix remodeling, and anti-fibrotic signaling^47^. While not discussed individually here, their collective involvement supports the notion that EPs are characterized by a multifaceted molecular landscape, distinct from that observed in UFs. Chiu et al.^5^ performed RNA sequencing on paired samples of EPs and adjacent endometrial tissue from infertile women, identifying 322 DEGs. Their analysis revealed significant dysregulation of Wnt signaling and vascular smooth muscle contraction pathways, with genes such as *DKK1* and *DKKL1* upregulated, and *GPC3*,* GREM1*,* RSPO3*,* SFRP5*, and *WNT10B* downregulated. Additionally, nearly all genes involved in vascular smooth muscle contraction, including *ACTA2*,* MYL9*, and *TAGLN*, were downregulated in polyps. Lin et al.^48^ employed both bulk and single-cell RNA sequencing to characterize transcriptional and cellular alterations in EPs compared to adjacent eutopic endometrium. Their analysis revealed a marked increase and activation of mast cells within polyps, accompanied by distinct transcriptional shifts. Through regulatory network analysis, WT1 was identified as a central transcription factor driving mast cell proliferation, with downstream dysregulation of WT1 target genes involved in cell growth and tissue remodeling. Pathare et al.^49^ conducted a large-scale GWAS meta-analysis involving over 25,000 women with female genital tract polyps, identifying ten significant genomic risk loci, including exonic variants in *PRIM1* and *COL17A1*, which are implicated in cellular proliferation. Several of the associated loci had previously been linked to endometrial cancer and UFs, suggesting shared genetic mechanisms underlying tissue overgrowth and neoplastic processes. Moreover, genetic correlation analysis revealed a negative association with sex hormone-binding globulin and strong phenotypic links to endometriosis, leiomyoma, and abnormal uterine bleeding, reinforcing the clinical relevance of these findings and their potential to inform future diagnostic and therapeutic strategies.

Despite the distinct transcriptomic profiles observed in UFs and EPs, a small group of shared genes was consistently differentially expressed in both conditions when comparing PET to NET samples. Although the number was insufficient for functional enrichment analysis, several of these genes are known to participate in stromal remodeling (*CNN1*,* ACTG2*)^50,51^, immune regulation (*HLA-G*)^52,53^, transcriptional control (*NONO*)^54^, and cell proliferation (*TOB1-AS1*)^55^. Their concurrent dysregulation across both pathologies points to shared molecular responses that could be triggered by structural disruption or local immune activity, regardless of the lesion’s origin. These common features seem to reflect a general pattern of tissue adaptation, where endometrial cells respond to pathological stimuli through similar regulatory pathways. This convergence, despite the distinct anatomical and etiological backgrounds of UFs and EPs, highlights the possibility that certain transcriptomic signatures may serve as broader indicators of uterine tissue reactivity, rather than being exclusive to a specific condition.

## Limitations

This study is exploratory in nature and subject to several limitations stemming from its clinical and methodological context. Although two distinct pathological entities were analyzed, both study groups represent uterine pathology. As such, the study lacks a strictly healthy control group. Due to ethical constraints, obtaining endometrial biopsies from asymptomatic women without clinical indications for hysteroscopy is not feasible, which limits the ability to define a baseline transcriptomic profile of unaffected endometrium. Importantly, NET should not be interpreted as biologically healthy endometrium, but rather as macroscopically non-lesional, pathology‑associated tissue sampled from uteri affected by benign uterine disease, which constrains direct comparisons to physiological baseline endometrial biology. Because endometrial gene expression is highly dynamic and responsive to hormonal fluctuations and menopausal status, uncontrolled variation in hormonal milieu represents an important source of biological variability that may contribute to the observed differences between groups, particularly in EP‑associated comparisons. To partially mitigate this confounding effect, transcriptomic analyses were restricted to premenopausal and perimenopausal women, although menstrual cycle phase and circulating hormone levels were not formally controlled at the time of sampling. An additional limitation of this study is the lack of racial and ethnic diversity in the cohort, as all participants were of Caucasian background. Because race‑dependent differences in UFs biology and transcriptomic profiles are well established, the findings related to UF‑associated endometrial tissue also may not be directly generalizable to other populations. At the same time, the ethnic homogeneity of the cohort may have reduced inter‑individual biological variability, increasing internal consistency of transcriptomic comparisons within this exploratory study. UFs are molecularly heterogeneous, encompassing distinct subtypes such as MED12‑mutant, HMGA2‑overexpressing, and FH‑deficient tumors; therefore, the lack of fibroid genotyping represents an important limitation of this study. Consequently, UF‑associated endometrial transcriptomic patterns cannot be resolved at the subtype level and should therefore be interpreted at the level of fibroid‑associated endometrial responses rather than subtype‑specific fibroid biology.

The sample size is modest, which may reduce statistical power and the ability to detect subtle gene expression differences. Nevertheless, the paired sample collection strategy, comparing pathologically altered and adjacent macroscopically non‑lesional endometrial tissue within the same uterus, helps reduce inter‑individual variability at the sampling level. Additionally, the use of bulk RNA sequencing captures an averaged transcriptomic signal across all cell types present in the tissue, which may mask cell‑type‑specific dysregulations. Differences in cellular composition between samples, including variable immune cell infiltration, may therefore contribute to the observed differential gene expression and cannot be disentangled without cell‑type‑resolved approaches such as single‑cell or spatial transcriptomics. While this limits biological resolution, bulk RNA sequencing remains a valid and widely used approach for initial, exploratory transcriptomic profiling of clinically obtained endometrial tissues. An additional technical limitation is the use of the hg19 reference genome for read alignment; although this genome build is supported by the Ion AmpliSeq™ Transcriptome v1 annotation, more recent genome assemblies (hg38) provide improved gene annotation, and subtle annotation‑dependent effects cannot be fully excluded.

## Conclusion

This study provides a detailed transcriptomic comparison of endometrial tissues associated with UFs and EPs, revealing distinct molecular profiles observed in UF‑ and EP‑associated endometrial tissue shaped by the anatomical origin and biological activity of each lesion. The identification of condition-specific and shared transcriptomic features in UF- and EP-associated endometrial tissue enhances our understanding of how benign uterine pathologies modulate the molecular landscape of the endometrium. Genes such as *IL17B*,* PTGS2*,* FOXP3*, and *DKK1*, among others, may represent candidate genes for future investigation into endometrial responses associated with benign uterine pathology. The study also highlights the importance of immune and stress-related signaling in the pathogenesis of both conditions, suggesting avenues for future research into targeted interventions. Furthermore, the integration of previous multi-omics and GWAS findings supports the notion that fibroids and polyps share genetic and molecular links with other gynecological disorders, including implantation failure and abnormal uterine bleeding. These insights may inform personalized approaches to diagnosis and treatment, particularly in reproductive medicine. Despite its exploratory nature and limitations, this study offers novel insights into the molecular landscape of endometrial tissue affected by UFs and EPs.

## Supplementary Information

Below is the link to the electronic supplementary material.

Supplementary Material 1. Fig. 1. Functional annotation of 3134 EP-associated genes revealed 194 enriched pathways across Biological Process (BP), Molecular Function (MF), and Cellular Component (CC) categories based on Gene Ontology (GO) analysis. Supplementary Table 1. Differentially expressed genes (DEGs; adjusted p-value < 0.05) in pairwise comparisons between uterine fibroids (UFs) and endometrial polyps (EPs) across two tissue types: macroscopically non‑lesional endometrial tissue (NET) and pathologically altered endometrial tissue (PET). The table includes: (1) NET: UFs vs. EPs, (2) PET: UFs vs. EPs, and (3) a Venn diagram illustrating unique and shared gene sets between comparisons. Supplementary Table 2. Gene Ontology (GO) and Reactome pathway enrichment analysis of 383 PET-specific genes using ClueGO. The table includes: (1) GO enrichment results across Biological Process (BP), Molecular Function (MF), and Cellular Component (CC) categories; and (2) Reactome pathway annotations. Supplementary Table 3. Differentially expressed genes (DEGs; adjusted p-value < 0.05) distinguishing pathologically altered endometrial tissue (PET) from macroscopically non‑lesional endometrial tissues (NET), analyzed separately for endometrial polyps (EPs) and uterine fibroids (UFs). The table includes: (1) EPs: PET vs. NET, (2) UFs: PET vs. NET, and (3) a Venn diagram showing shared and unique DEGs between EP and UF comparisons. Supplementary Table 4. Functional enrichment analysis of differentially expressed genes (DEGs) distinguishing pathologically altered endometrial tissue (PET) from macroscopically non‑lesional endometrial tissues (NET), focusing on gene sets unique to endometrial polyps (EPs) and uterine fibroids (UFs). The table includes: (1) GO pathway mapping of 3134 EP-associated genes across Biological Process (BP), Molecular Function (MF), and Cellular Component (CC) categories; (2) GO immune-related pathways enriched in the analyzed gene set; and (3) Reactome pathway enrichment results (adjusted p-value < 0.05) based on EP-associated genes.

## Data Availability

The datasets presented in this study can be found in online repositories. The names of the repository and accession number can be found below: https://www.ncbi.nlm.nih.gov/bioproject/1347239.

## Abbreviations

UFs: Uterine fibroid(s)
EPs: Endometrial polyp(s)
PET: Pathological endometrial tissue
NET: Adjacent macroscopically normal endometrial tissue
GWAS: Genome-wide association study
RNA-Seq: RNA sequencing
DEGs: Differentially expressed gene(s)
GO: Gene ontology
BP: Biological process
MF: Molecular function
CC: Cellular component
padj: Adjusted p-value
